# Bacterial Flora and Treatment Strategies in Women With Escherichia coli Urinary Tract Infections

**DOI:** 10.7759/cureus.56552

**Published:** 2024-03-20

**Authors:** Saisri Mahesh, Durai Singh Carmelin, Raman Muthusamy

**Affiliations:** 1 Center for Global Health Research, Saveetha Dental College and Hospitals, Saveetha Institute of Medical and Technical Sciences, Chennai, IND; 2 Center for Global Health Research, Saveetha Institute of Medical and Technical Sciences, Chennai, IND

**Keywords:** treatment strategies, multiplex pcr, antibiotic resistance (abr), e.coli, urinary tract infections

## Abstract

Introduction

This study explores the intricate relationship between bacterial flora and the occurrence of *Escherichia coli *(*E. coli*) infections in gynecological patients. It aims to provide insights into the various treatment strategies used to effectively manage bacterial pathogens, especially *E. coli* infections. By conducting a comprehensive analysis of the bacterial flora in gynecological patients, the study highlights the notable presence of *E. coli*, prompting further investigation into the factors that contribute to its colonization. The objective of the study is to comprehensively investigate and detect urinary tract infections (UTIs) specifically caused by *E. coli *among gynecological patients. The study aims to delve into bacterial flora prevalence, antibiotic resistance patterns, and potential virulence factors. Through this analysis, the study intends to identify effective strategies for rapid detection and diagnosis of UTIs caused by *E. coli* by utilizing advanced microbiological and molecular techniques. Furthermore, the study aims to formulate and propose a strategic treatment approach with a particular emphasis on selecting appropriate antibiotics to reduce the risk of severe infections and associated complications.

Materials and methods

The methodology employed in this study included the isolation and characterization of bacterial strains from clinical samples obtained from gynecological patients. A total of 52 urine specimens were collected from patients with complaints of infection in the urinary tract and infertility. These samples underwent both preliminary and confirmatory microbiological analysis, such as gram staining, biochemical confirmation test, and antibiotic susceptibility, and further proceeded with the multiplex polymerase chain reaction (PCR) technique. The results of PCR and antibiotic susceptibility revealed the specific gene involvement and resistant characteristics of *E. coli*.

Results

The findings revealed a total of 32 specimens positive for *E. coli,* of which 10 patients had infertility complaints and 22 patients had UTIs. The preliminary test, gram staining, showed the gram-negative bacilli *E. coli*, and the nutrient agar plate revealed smooth circular translucent colonies; MacConkey agar showed pink-colored lactose-fermented colonies; and the blood and chocolate agar plates showed grayish white moist gamma-hemolytic colonies. The biochemical confirmation of *E. coli *resulted in positive for indole and methyl red tests and negative for Voges-Proskauer and citrate utilization tests. The multiplex PCR analysis confirmed the *E. coli* strains with the presence of two target genes, *stx2d *and* stx2e*.

Conclusion

To summarize, this study offers valuable insights into the bacterial flora of gynecological patients impacted by *E. coli* infections, which provides a foundation for the development of precise and efficient treatment strategies. The results emphasize the importance of personalized treatment approaches that consider both the microbiological characteristics of the infection and the evolving landscape of antibiotic resistance. The implication of this research extends to enhancing clinical outcomes and alleviating the burden of *E. coli* infections in gynecological settings.

## Introduction

The typical composition of the vaginal microflora encompasses a range of five to fifteen distinct bacterial species, comprising both aerobic and anaerobic organisms. This diverse microbial community has the capacity to give rise to various clinical diseases. Among the prevalent constituents of this microflora is *Escherichia coli*, a gram-negative facultative anaerobe [1]. This species can also be commensal, as they are normal intestinal microbial flora; however, they can also be a cause of various infectious diseases [2]. The barrier distinguishing commensalism from virulence emerges from the intricate balance involving the host’s condition, existence, and activation of virulence factors within the bacteria [3]. While the majority of *E. coli* strains are considered harmless, certain pathogenic variants, such as *E. coli* O157:H7, have the potential to induce severe infections, including urinary tract infections (UTIs) and complications leading to infertility [4]. Pathogenic strains of *E. coli* produce toxins and other virulence factors capable of causing damage to host cells. These pathogenic characteristics are encoded by the genes within the pathogenic bacteria. Various types of infections occur based on the microbial pathogen’s region of existence and level of microbial count and virulent factors [5]. The presence of *E. coli* is commonly found in the vaginal region of both pregnant and non-pregnant women. This colonization represents the significant precursor in the cascade leading to the development of various infections, including urinary tract infections and infertility. While UTIs affect individuals of both sexes, those that lead to infertility are more prevalent in males than females. This is due to various factors, including personal hygiene practices, catheter usage, and extensive antibiotic use, which can contribute to the development of infertility conditions. The transmission of this infection is characterized by the transfer of bacteria from the fecal source to the vaginal region, which then extends to the urinary and neonatal domains [6]. Vaginal infections constitute the cause for primary healthcare consultations globally, yet their occurrence and underlying causes exhibit variations among different populations. Accurate clinical diagnosis is essential to prescribe an effective therapeutic approach for these infections [7].

The management of cases involving urinary tract infections (UTIs) has traditionally relied on antibiotic therapy. However, the elevated prevalence of antibiotic resistance in *E. coli* associated with clinical infections has led to prolonged and more severe disease. Recent epidemiological studies indicate a substantial resistance ranging from 50% to 100% among the *E. coli* strains to commonly used antibiotics. These antibiotics include tetracycline, cefotaxime, gentamicin, ampicillin, cotrimoxazole, norfloxacin, and cephalothin [8-10]. The objective of the study is to ascertain gynecological infections attributed to *E. coli *and develop a therapeutic approach for affected patients.

## Materials and methods

Nutrient agar, MacConkey agar, blood agar, chocolate agar, biochemical reagents, Muller-Hinton agar, antibiotic discs, and gram stain reagents were purchased from Hi-Media (Mumbai, India). A standard *E. coli *strain was obtained from the Department of Microbiology at Saveetha Medical College.

Sample collection

To progress with this study, 52 mid-stream urine specimens were collected from female patients with gynecological infections like UTI and infertility who attended Saveetha Medical College and Hospitals. The samples were further cultured in the medium, and *E. coli* strains were confirmed by the biochemical tests. Following all the stipulated ethical guidelines, ethical clearance was obtained from the Institutional Review Board and Ethics Committee (IRB No. 112101140).

Methodology

Isolation of Test Organism

Totally 52 mid-stream urine specimens were obtained from female patients with clinical indications of UTI infection and infertility. Nutrient agar and MacConkey agar plates were prepared for the cultivation of microbial growth. Aseptically, urine samples were streaked onto nutrient agar and MacConkey agar plates and then incubated for 18-24 hours at 37°C. Subsequent to incubation, the morphology of the bacteria was observed by gram staining. As selective media, blood agar and chocolate agar plates were prepared and subjected to microbial growth. Furthermore, biochemical tests, including the indole, methyl red, Voges-Proskauer, and citrate utilization tests, collectively known as the IMVIC tests, were performed to confirm the presence of Enterobacteriaceae family members, specifically *Escherichia coli *(*E. coli*) [10].

Testing for antimicrobial susceptibility of *E. coli* isolates

An antimicrobial susceptibility test was conducted based on the Kirby Bauer disc diffusion method to assess resistance (R%) [11]. The following antibiotics were examined: erythromycin (10 µg), amoxicillin (10 µg), tetracycline (10 µg), nitrofurantoin (10 µg), and gentamicin (10 µg). These antibiotics were selected based on the prescription patterns observed at the study site. Subsequently, a loopful of bacterial isolates from the cultured colony was transferred into a tube containing nutrient broth, which was then swabbed onto Muller-Hinton agar (MHA) plates using a sterile cotton swab. The antibiotic discs were placed on the agar plates and incubated for 24 hours at 37°C. Antimicrobial activity was determined by measuring the zones of inhibition, and the collected data were analyzed using Microsoft Office Excel (Microsoft Corporation, Redmond, Washington, USA). Significant differences between samples were assessed, with p-values below 0.005 (p<0.005) considered statistically significant [12].

Molecular profiling of *E. coli *isolates

A polymerase chain reaction (PCR) was employed for additional identification of the test organism. The stages involved are DNA extraction, PCR, and gel electrophoresis. Genomic DNA extraction was performed by following the protocol outlined by Magray et al. [13]. 1.5 mL of the cultured organism from the broth sample was taken in an Eppendorf tube and centrifuged at 10,000 rpm for five minutes. Following centrifugation, the supernatant was discarded, and the pellets underwent two washes with sterile water. Subsequently, pellets were added to 200 µL of sterile water, followed by vortexing for homogenization. Followed by vortexing and centrifugation at 12,000 rpm for five minutes, the mixture was kept in a dry bath at 100°C for 10 minutes. Finally, the supernatant contains the DNA, which was transferred to another tube and stored at −20°C.

Polymerase chain reaction technique (PCR)

To perform the PCR technique, the required reagents, such as Taq buffer, MgCl_2_, dNTPs, forward primer, reverse primer, distilled deionized water, and genomic DNA, were made up to a volume of 15 µL. The prepared sample, comprising the requisite reagents, was loaded onto the PCR machine. The cycling parameters were established, commencing with an initial denaturation at 95°C for five minutes. Subsequently, a repetitive cycle was implemented 35 times, featuring denaturation at 94°C for 60 seconds, primer annealing at 60°C for 90 seconds, and elongation or extension at 72°C for 90 seconds. This study developed a multiplex PCR assay using two primer sets to detect genes related to *E. coli* attributes, including the virulence genes *stx2d *and *stx2e* associated with diseases. Specific primers were included for amplifying *E. coli* as an internal positive control. Validation was done using individual primer-based PCR [10].

Gel electrophoresis method 

A 1.5% agarose gel was prepared by dissolving 1.5 g of agarose in 100 mL of Tris acetate Ethylene Diamine Tetra Acetic Acid (EDTA) buffer and cooling the solution. Ethidium bromide (4 µL) was added, and the mixture was poured into a casting apparatus with a comb to form wells, allowed to solidify, and loaded with PCR amplicon matrices. Electrophoresis was conducted at 340 V for 30 minutes. A DNA ladder was used as a reference standard for quantifying base pairings during gel electrophoresis [14]. The purity of the DNA was assessed using a nanodrop spectrophotometer. The selected target genes, forward and reverse primer sets, are mentioned in the tabular column below. The amplicon size obtained from the multiplex primer sets matched precisely with the sizes predicted with the reference of Wang et al. (Table 1).

**Table 1:** Primers used in E. coli detection of urinary tract infection. F: forward primer, R: reverse primer.

Primer set	Primers	Sequence (5’to 3’)	Target gene	Location within gene	Amplicon size (bp)	Genbank accession no.
A	Stx2e-a, Stx2e-b	F:ATGAAGTGTATATTGTTAAAGTGGA	stx2e	204-228 506-485	303	M36727
		R:AGCCACATATAAATTATTTCGT				
B	Stx2d-a, Stx2d-b	F:GGTAAAATTGAGTTCTCTAAGTAT	stx2d	1221–1244 1395–1375	175	AF043627
		R:CAGCAAATCCTGAACCTGACG				

## Results

**Figure 1:**
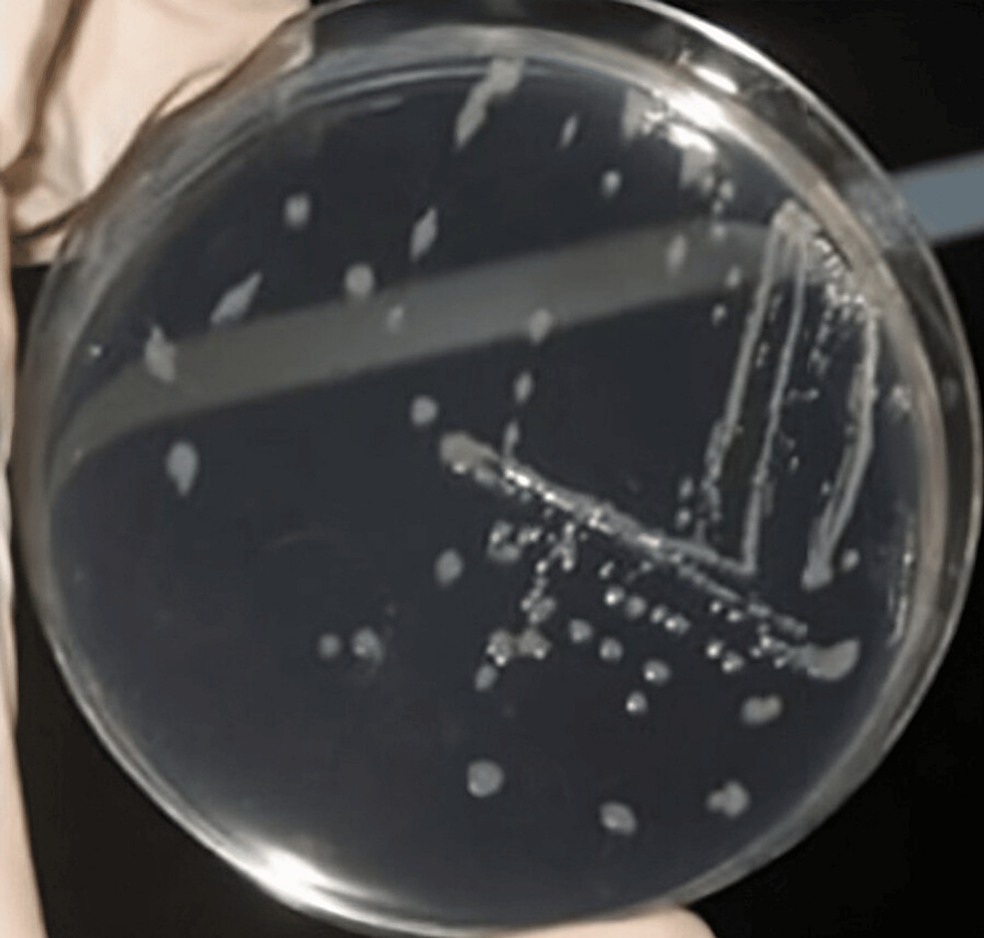
Colony morphology of E. coli in nutrient agar plate.

**Figure 2:**
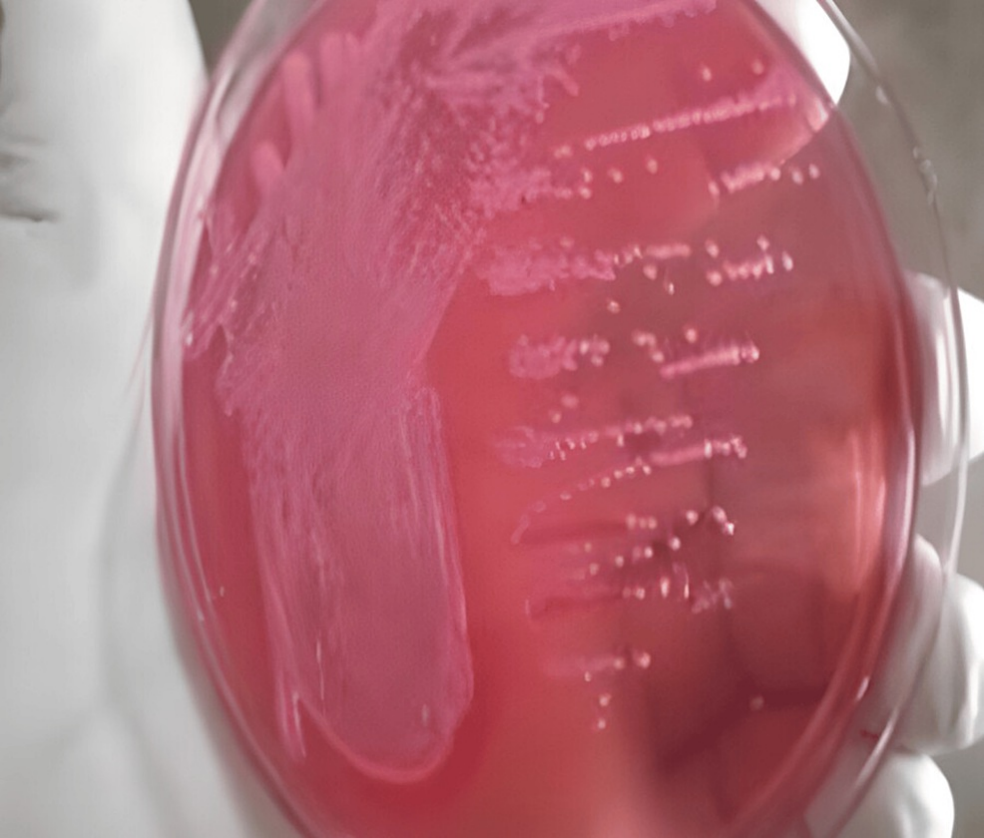
Colony morphology of E. coli in MacConkey agar plate.

**Figure 3:**
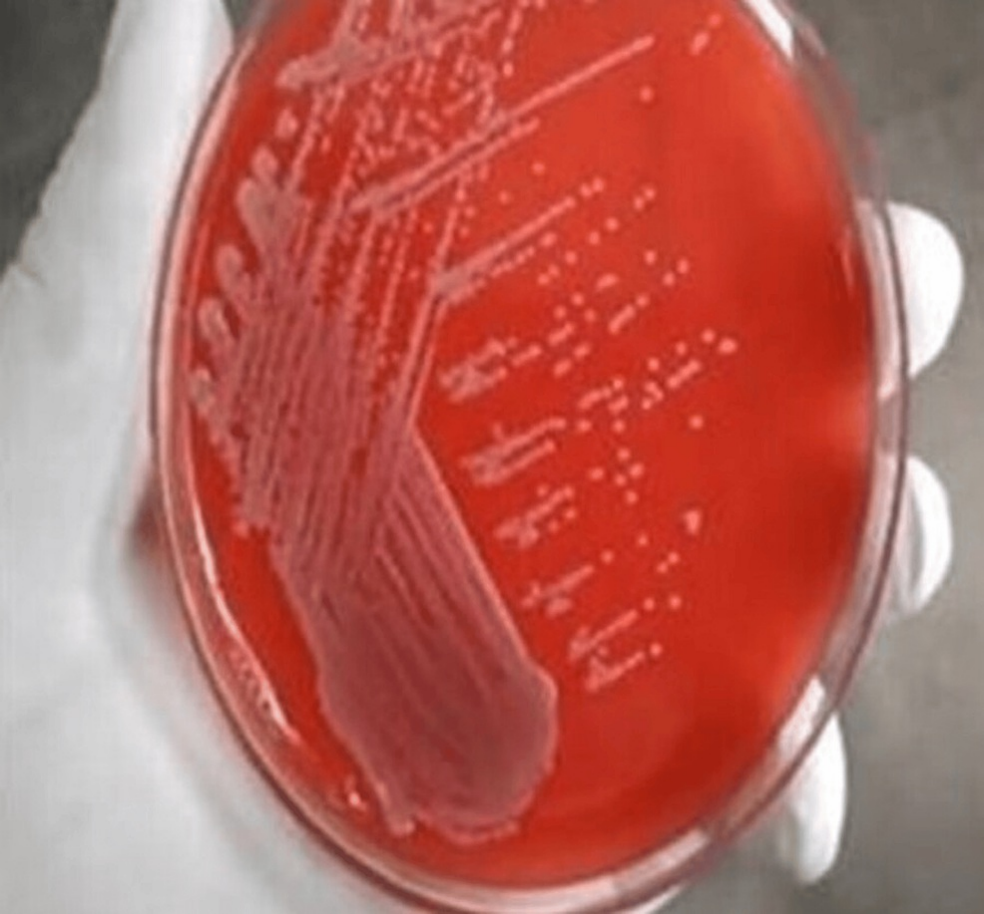
Colony morphology of E. coli in blood agar plate.

**Figure 4:**
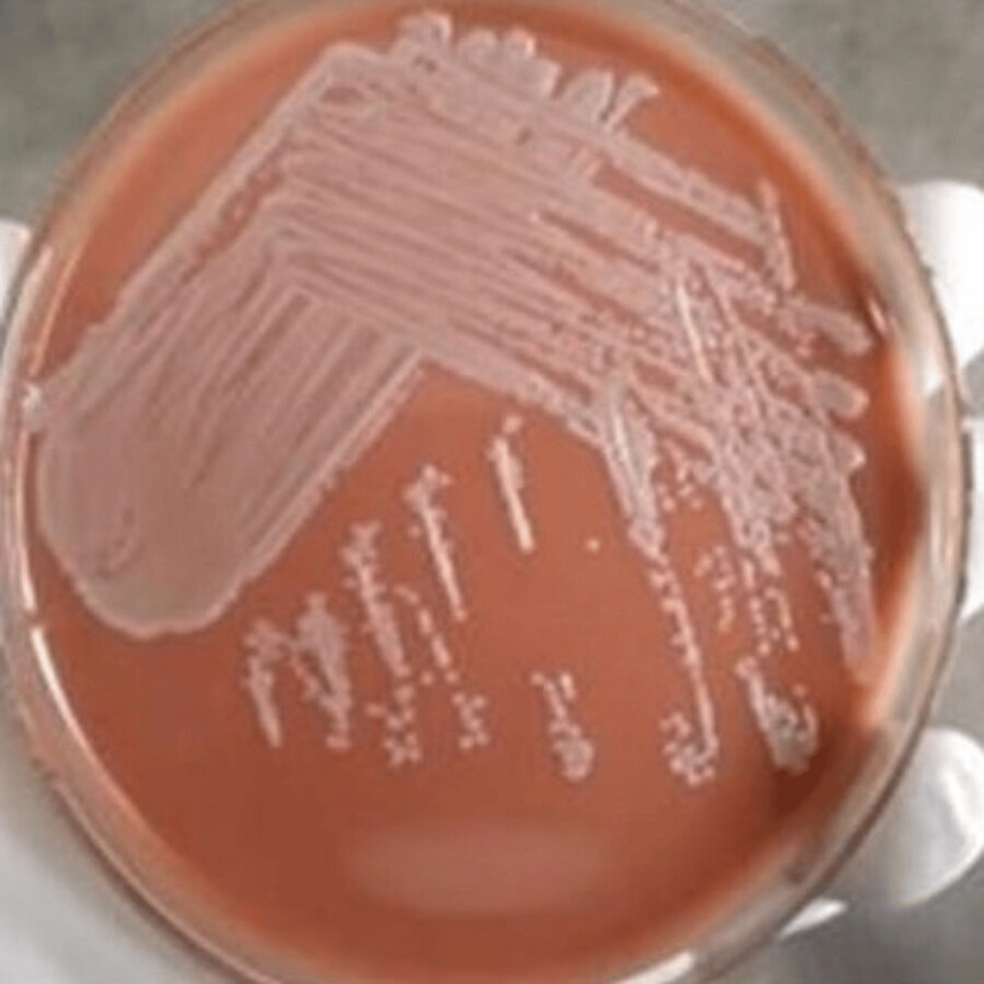
Colony morphology of E. coli in chocolate agar plate.

Out of the 52 urine specimens collected from female patients, 32 specimens tested positive for *E. coli*, with 10 specimens obtained from patients diagnosed with infertility conditions and 22 specimens from patients diagnosed with UTI. Additionally, 20 specimens were found to be positive for other types of microbes, such as *Klebsiella *and *Proteus *(Table 2). Based on the culture growth colony characteristics of *E. coli *observed in the Gram staining revealed gram-negative bacilli (Table 3), nutrient agar, MacConkey agar, blood agar, and chocolate agar plates, the morphology of the colonies showed smooth, circular, and translucent colonies in nutrient agar plates (Figure 1). Pink-colored lactose-fermented colonies were observed on the MacConkey agar plate (Figure 2), and grayish-white moist, gamma-hemolytic colonies were observed on blood agar and chocolate agar plates (Figures 3, 4).

**Table 2:** Data of sample collection and positive samples for E. coli infection. UTI: urinary tract infection.

Total number of urine specimens collected	Positive for *E. coli*	Patients with infertility condition (positive for *E. coli*)	Patients with UTI (positive for *E. coli*)	Positive for other bacterial infection
52	32	10	22	20

**Table 3:** Colony morphology of the preliminary test performed.

Tests performed	Colony morphology
Preliminary test (Gram staining)	Gram-negative rod-shaped bacteria
Agar plate method	
Nutrient agar	Smooth circular translucent colonies
MacConkey agar	Pink-colored lactose-fermented colonies
Blood agar	Grayish-white moist, gamma-hemolytic colonies
Chocolate agar	Grayish-white moist, gamma-hemolytic colonies

Biochemical reactions of the organism

Confirmation of *E. coli* was achieved by observing positive results in biochemical reactions, such as indole and methyl red, indicating the organism's ability to produce indole from tryptophan and maintain stable acid end products from glucose fermentation, respectively. Conversely, the Voges-Proskauer and citrate utilization tests showed negative results, indicating the organism's inability to utilize the butylene glycol pathway and citrate as a sole carbon source, respectively. These findings collectively confirm the identification of the organisms as *E. coli *(Table 4).

**Table 4:** Biochemical confirmation test results.

Indole	Methyl red	Voges-Proskauer	Citrate utilization
Positive	Positive	Negative	Negative

Multiplex PCR for the detection of *E. coli* virulence genes

**Figure 5:**
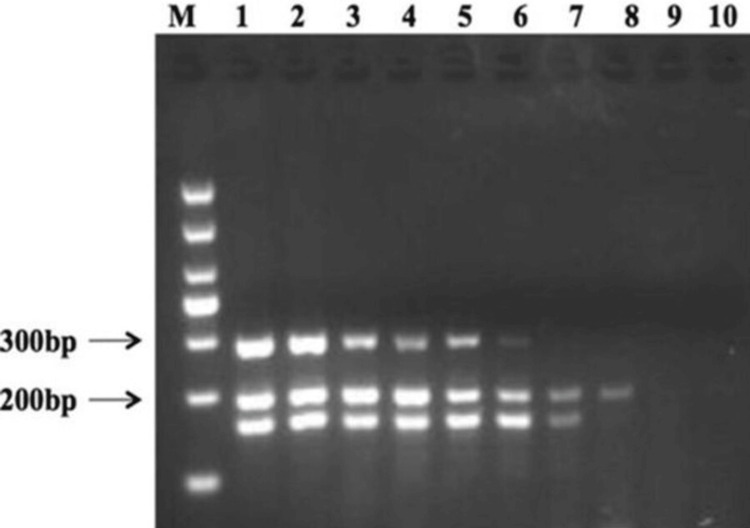
Comparison of the DNA band with the ladder by gel electrophoresis. bp-base pairs.

To validate the effectiveness of the multiplex PCR technique, 32 strains of *E. coli *were individually screened for toxin genes using previously described methods. Out of the 52 samples collected, 32 tested positive for *E. coli*, along with other bacterial strains. Among these, 10 samples were obtained from patients with infertility complaints, while 22 samples were from patients with UTIs. From the 32 positive samples, five samples each were selected from infertile patients and UTI patients for PCR analysis. During the PCR analysis, wells 9 and 10 showed negative results, while wells 7 and 8 exhibited a low band intensity, indicating a lower load of the *E. coli* genome. The reaction conditions for the multiplex PCR assays were cautiously optimized to ensure successful amplification of all target gene sequences. The primers were specifically designed to target coding regions within the gene structure encoding the *stx2* group of toxins. Each primer set utilized in the assay shared an identical annealing temperature, minimizing the risk of nonspecific amplification. During agarose gel electrophoresis analysis, the reference *E. coli *strain (positive control) served as a template in the multiplex PCR primer sets, aiding in confirming the presence of amplified product profiles. Sets A and B resulted in two bands when analyzing a mixture of DNA extracts from the respective strains carrying genes stx2e and stx2d, with sizes of 300 bp and 200 bp, respectively (Figure 5). The amplicon sizes obtained from the multiplex primer sets aligned with the predicted sizes based on primer design, confirming the existence and quality of *E. coli* DNA amplification and validating the PCR conditions.

Antimicrobial resistance of *E. coli*

The antimicrobial resistance profiles of the 32 positive specimens were assessed against respective antibiotics, including erythromycin, amoxicillin, tetracycline, nitrofurantoin, and gentamycin. *E. coli *strains isolated from females with UTIs exhibited antimicrobial resistance to erythromycin (n=22) (27.27%), amoxicillin (n=22) (22.72%), nitrofurantoin (n=22) (27.27%), tetracycline (n=22) (13.63%), and gentamycin (n=22) (9.09%). Similarly, specimens obtained from females with infertility conditions displayed antimicrobial resistance to erythromycin (n=10) (30%), amoxicillin (n=10) (20%), nitrofurantoin (n=10) (30%), tetracycline (n=10) (10%), and gentamycin (n=10) (10%). Overall, the results indicated that *E. coli *was highly sensitive to erythromycin, amoxicillin, nitrofurantoin, and tetracycline (Table 5).

**Table 5:** Antimicrobial resistance of E. coli isolates. n: number of samples collected, R%: antibiotic drug resistance in percent, UTI: urinary tract infection.

*E. coli* isolates types (n=32)	Antibiotics				
	Erythromycin (R%)	Amoxicillin (R%)	Nitrofurantoin (R%)	Tetracycline (R%)	Gentamycin (R%)
Isolated from female with UTI (n=22)	6 (27.27%)	5 (22.72%)	6 (27.27%)	3 (13.63%)	2 (9.09%)
Isolated from female with infertility (n=10)	3 (30%)	2 (20%)	3 (30%)	1 (10%)	1 (10%)

## Discussion

The confirmation tests conducted in this study provide compelling evidence for the presence of *E. coli*. Gram staining, a fundamental microbiological technique, revealed the characteristics and morphology of gram-negative pink-colored rod-shaped bacilli, which is consistent with the typical appearance of *E. coli* under the microscope. This finding is crucial as it establishes the identity of the bacterial species present in the samples. Furthermore, the results observed on MacConkey agar plates, showing lactose-fermenting pink colonies, further support the presence of *E. coli*. MacConkey agar is a selective and differential medium commonly used to isolate and differentiate lactose-fermenting gram-negative bacteria, such as *E. coli*, from non-fermenters. The detection of lactose fermentation by *E. coli* colonies on MacConkey agar is indicative of its ability to utilize lactose as a carbon source, which is a characteristic feature of this bacterium [15]. Other similar studies of blood agar and chocolate agar showed the same grayish-white moist colonies [16]. Table 4 illustrates the diverse biochemical reactions conducted, revealing that the organisms demonstrated the capability to produce indole from tryptophan with the addition of Kovac’s reagent [17]. In the methyl red test, the results indicate that the organisms produced and sustained stable acid end products during glucose fermentation in the glucose broth. The results of the Voges-Proskauer test revealed that the organism did not exhibit utilization of the butylene glycol pathway; similarly, in the citrate utilization test, it was observed that the organisms could not utilize citrate as the sole carbon source [18]. When compared to the other studies, the obtained strains of *E. coli *showed similar results, which confirms the presence of the gram-negative bacilli bacteria *E. coli*. Overall, the results of these biochemical tests provide valuable insights into the metabolic capabilities of the organism, aiding in its identification and characterization. In this study, the negative results of the Voges-Proskauer test and citrate utilization test further support the identification of the organism as *E. coli*, as these characteristics are consistent with the known metabolic profile of this species.

Certain strains of *E. coli* exhibit an elevated level of virulence toward humans, indicating that they possess characteristics that make them more harmful to health. These virulent strains often carry genetic elements such as pathogenicity islands, plasmids, or bacteriophages encoding virulence factors, which enable them to cause more severe and potentially life-threatening diseases. One of the most well-known virulence factors associated with pathogenic *E. coli* strains is the production of Shiga toxins (Stx), also known as verotoxins. These toxins are responsible for the development of severe clinical conditions, such as hemorrhagic colitis and hemolytic uremic syndrome (HUS), which can lead to kidney failure and even death. *E. coli *strains that produce Shiga toxins are often referred to as Shiga toxin-producing *E. coli* (STEC) or enterohemorrhagic *E. coli* (EHEC). Other virulent factors commonly associated with the pathogenic E. coli strains include adhesins, which allow the bacteria to adhere to colonize the epithelial cells of the gastrointestinal tract or urinary tract. Once colonized, these bacteria can produce toxins or other factors that damage host tissues and contribute to the development of disease. Furthermore, certain strains of *E. coli* may possess antibiotic resistance genes, which can confer resistance to commonly used antibiotics. This not only complicates the treatment of infections caused by these strains, but also increases the risk of treatment failure and the spread of antibiotic-resistant bacteria within the community [19]. Diagnostic tools, such as PCR techniques, are utilized to detect specific genes responsible for virulent factors.

Multiplex PCR was employed in this study to detect the presence of specific target genes, namely, *stx2e *and *stx2d*. The application of multiplex PCR analysis to delineate the diverse subtypes of the *stx2* genes has been extensively documented [20]. This methodology involves the simultaneous amplification of multiple target DNA sequences, allowing for a comprehensive characterization of the specific subtypes within the *stx2* gene family. This approach provides a detailed and thorough understanding of the genetic variations present, contributing to the exploration of the microbial landscape under investigation [21-23]. *E. coli *Shiga toxin* *(Stx) toxins are broadly categorized into two main groups: *stx1* and *stx2*, in which *stx1* represents a relatively uniform group of toxins that exhibit similarity with the Shiga toxins, implying structural and functional similarities. The homogeneity within the *stx1* family underscores a more consistent and defined set of characteristics, aiding in the understanding of their biological properties and potential implications in microbial pathogenesis [22]. Apart from serological differences, the *stx2* group of toxins may exhibit variations in their in-vitro or in-vivo characteristics [24]. In-vitro studies, encompassing antibiotic susceptibility testing, will be conducted alongside in-vivo investigations involving animal studies.

Antibiotic treatments are controversial in certain circumstances, like the presence of the *stx* gene in *E. coli*, which indicates potential toxin production and induces an increased release of toxins, exacerbating the illness. The absence of the *stx* gene suggests a milder course. Hence supportive care, including fluid electrolyte replacement and dialysis in complicated conditions [25]. Certain relevant studies say that nitrofurantoin is said to be the first-line drug, and at present, its resistance is very low [26]. Studies reported that nitrofurantoin 50 mg, taken once daily for a duration of six months, emerges as the top choice concerning effectiveness, cost-effectiveness, and minimal adverse effects for chemotherapeutic strategies in UTIs [27]. The amoxicillin-clavulanic acid and gentamicin were also recommended as the first-line therapy for UTI [28]. For severe UTI, empirical treatment should commence upon diagnosis. In cases where the symptoms and mild treatment can be guided by the results of genomic identification [29]. Understanding the differential abundance of these genes contributes valuable insights into the genetic diversity and potential pathogenicity of the *E. coli* strains being analyzed, and the initiation of possible treatment within a short span of time should be implemented to avoid the increased infection rate. Personal hygiene and cleanliness also have to be followed by individuals to get rid of severe infections.

Limitations

In the present study, a limited number of samples were analyzed for antimicrobial resistance, which may have restricted the breadth of insights gained regarding bacterial flora and resistance patterns. Expanding the sample size to include a more diverse range of samples, such as vaginal douches, swabs, and high vaginal swabs, could provide a more comprehensive understanding of the microbial population and their resistance profiles. Additionally, incorporating a wide array of antimicrobial agents, including both first-line and second-line drugs, would offer a more nuanced assessment of resistance patterns among *E. coli* strains. Furthermore, the study primarily focused on specific target genes in *E. coli*, which may have limited the scope of antimicrobial resistance analysis. Given the complexity of UTIs and infertility in women, which can often present with overlapping symptoms and underlying reproductive health issues, such as sexually transmitted infections or pelvic inflammatory disease (PID), a broader approach to antimicrobial resistance testing is warranted. UTIs and infertility in women may be multifactorial in nature, involving not only bacterial infections but also various reproductive health factors, such as endometriosis or tubal blockages. Therefore, future investigations should aim to expand the identification of antibiotic resistance patterns across a wider range of samples and antimicrobial agents. Additionally, comprehensive diagnostic approaches should be employed to differentiate between various conditions contributing to UTIs and infertility in women. This will facilitate the development of more targeted and effective interventions tailored to the specific microbial and reproductive health challenges faced by women experiencing these conditions.

## Conclusions

In conclusion, the study on the bacterial flora and treatment strategy among gynecological patients with *E. coli* infections provides valuable insights into the management of these cases. The analysis of bacterial flora, including the identification of specific strains such as* E. coli*, contributes to a better understanding of the microbial landscape in gynecological infections. Moreover, the utilization of advanced techniques like multiplex PCR, and analysis enhances the accuracy of microbial identification and allows for more detailed characterization of genetic markers. This study underscores the necessity of a comprehensive approach to addressing *E. coli* infections in gynecological patients, considering both bacterial diversity and the evolving landscape of antibiotic resistance. The findings not only support the development of targeted and effective treatment strategies but also emphasize the significance of ongoing research to stay ahead of emerging challenges in the field of gynecological infections. Continued efforts in understanding bacterial flora and refining treatment approaches are essential for improving patient outcomes and mitigating the impact of infections caused by *E. coli* in gynecological settings.
